# Surgery for Coagulopathy-Related Intracerebral Hemorrhage: Craniotomy vs. Minimally Invasive Neurosurgery

**DOI:** 10.3390/life11060564

**Published:** 2021-06-15

**Authors:** Yen-Bo Liu, Lu-Ting Kuo, Chih-Hao Chen, Woon-Man Kung, Hsin-Hsi Tsai, Sheng-Chieh Chou, Shih-Hung Yang, Kuo-Chuan Wang, Dar-Ming Lai, Abel Po-Hao Huang

**Affiliations:** 1 Division of Neurosurgery, Department of Surgery, National Taiwan University Hospital, College of Medicine, National Taiwan University, Taipei 10617, Taiwan; imountainpig@ntuh.gov.tw (Y.-B.L.); ltkuo@ntuh.gov.tw (L.-T.K.); swonyang@ntuh.gov.tw (S.-H.Y.); wang081466@ntuh.gov.tw (K.-C.W.); laidd@ntuh.gov.tw (D.-M.L.); 2 Department of Neurology, National Taiwan University Hospital, College of Medicine, National Taiwan University, Taipei 10617, Taiwan; moulinrouge@ntuh.gov.tw (C.-H.C.); hsinhsi@ntuh.gov.tw (H.-H.T.); 3 Department of Exercise and Health Promotion, College of Kinesiology and Health, Chinese Culture University, Taipei 11114, Taiwan; nskungwm@yahoo.com.tw; 4 Department of Neurology, National Taiwan University Hospital Bei-Hu Branch, Taipei 10617, Taiwan; 5 Division of Hematology, Department of Internal Medicine, National Taiwan University Hospital, College of Medicine, National Taiwan University, Taipei 10617, Taiwan; choushengchieh@ntuh.gov.tw

**Keywords:** intracerebral hemorrhage, coagulopathy, minimally invasive neurosurgery, comparison of surgical outcomes

## Abstract

Coagulopathy-related intracerebral hemorrhage (ICH) is life-threatening. Recent studies have shown promising results with minimally invasive neurosurgery (MIN) in the reduction of mortality and improvement of functional outcomes, but no published data have recorded the safety and efficacy of MIN for coagulopathy-related ICH. Seventy-five coagulopathy-related ICH patients were retrospectively reviewed to compare the surgical outcomes between craniotomy (*n* = 52) and MIN (*n* = 23). Postoperative rebleeding rates, morbidity rates, and mortality at 1 month were analyzed. Postoperative Glasgow Outcome Scale Extended (GOSE) and modified Rankin Scale (mRS) scores at 1 year were assessed for functional outcomes. Morbidity, mortality, and rebleeding rates were all lower in the MIN group than the craniotomy group (8.70% vs. 30.77%, 8.70% vs. 19.23%, and 4.35% vs. 23.08%, respectively). The 1-year GOSE score was significantly higher in the MIN group than the craniotomy group (3.96 ± 1.55 vs. 3.10 ± 1.59, *p* = 0.027). Multivariable logistic regression analysis also revealed that MIN contributed to improved GOSE (estimate: 0.99650, *p* = 0.0148) and mRS scores (estimate: −0.72849, *p* = 0.0427) at 1 year. MIN, with low complication rates and improved long-term functional outcome, is feasible and favorable for coagulopathy-related ICH. This promising result should be validated in a large-scale prospective study.

## 1. Introduction

A significant increase worldwide in patients with coagulopathy-related intracerebral hemorrhage (ICH) has been observed due to the increased use of antithrombotic agents over the last decade for the prevention of cardioembolic events, coronary heart disease, and thrombotic-related disease [1,2]. It is estimated that 10–13% and 27–30% of ICH patients who underwent surgery were on the treatment of anticoagulant agents and antiplatelet agents, respectively [3,4]. The use of vitamin K antagonists (VKAs) has been associated with ICH rates of up to 1.8% per annum, while ICH rates among novel oral anticoagulant (NOAC)-treated patients are 40–70% lower than that with warfarin [5]; of particular concern is the finding that Asians are four times more likely than Caucasians to develop VKA-related ICH [6]. However, the influence of antithrombotic agents on ICH expansion or neurologic outcomes remains controversial [3,7,8].

In addition to antithrombotic agents, the prevalence of end-stage renal disease (ESRD) among patients receiving hemodialysis in Taiwan is the highest in the world. ICH patients on hemodialysis are exposed to a latent activation of coagulation resulting in an elevated thrombogenic risk, which has been associated with an increased mortality rate ranging from 43.8 to 83% [9,10,11,12].

Rapid reversal of coagulopathy may help to limit ICH expansion and improve clinical outcomes [13]; however, approximately 9.5–33% of these patients will still require neurosurgical intervention, for whom scant evidence exists as to appropriate surgical management [14,15,16,17]. Recent studies have shown promising results with minimally invasive neurosurgery (MIN) in the reduction of mortality and improvement of functional outcomes in ICH patients [18,19,20]. We therefore retrospectively reviewed our surgical series of coagulopathy-related ICH patients and compared surgical outcomes following traditional craniotomy and MIN.

## 2. Materials and Methods

### 2.1. Subjects

We retrospectively analyzed data from 2013 to 2018 at National Taiwan University Hospital (NTUH), Taipei, Taiwan. Patients with coagulopathy who underwent surgery for ICH with preoperative head computed tomography (CT) and a follow-up head CT within 24 h after the surgery were included. These patients were not randomly assigned to receive different surgeries. Coagulopathy was defined as (1) using antiplatelet or anticoagulant agents, (2) platelet count < 100 k/μL, (3) international normalized ratio (INR) > 1.20, or (4) ESRD on hemodialysis. We excluded patients with vascular lesions (i.e., cerebral aneurysm, arteriovenous malformation, cavernous malformation), cerebellar ICH, traumatic ICH, tumor bleeding, hemorrhagic transformation after ischemic stroke, postoperative ICH, or coagulopathy-related ICH occurring in the intensive care unit (ICU) because these events were often related to systemic diseases, autoimmune dysfunction, acute infection, organ dysfunction, or disseminated intravascular coagulopathy. Patients whose primary treatment was external ventricular drainage (EVD) or decompressive craniectomy were also excluded. This study was approved by the Institutional Review Board of NTUH (approval number: 201611058RINA) and conducted in accordance with the applicable local regulations and the Declaration of Helsinki. Written informed consent was waived due to the use of secondary data.

### 2.2. Surgery Selection

There has been no consensus about the selection of surgical method in coagulopathy patients who require surgical removal of ICH. Even for spontaneous ICH, there are many different methods. In general, neurosurgeons determine the surgical approach (i.e., MIN or craniotomy) according to the location of ICH, type of ICH, and associated comorbidities.

Our consensus for surgical treatment of ICH included (1) a putaminal ICH with a hematoma volume greater than 30 mL, (2) a thalamic ICH with a hematoma volume greater than 20 mL and IVH with acute hydrocephalus, or (3) a subcortical hemorrhage greater than 30 mL with significant mass effect (midline shift greater than 5 mm and effacement of perimesencephalic cistern) [21].

Five major MIN techniques, namely stereotactic thrombolysis, craniopuncture, endoscopic, endoscope-assisted, and endoport-mediated, have been discussed previously [22]; we preferred the endoscope-assisted technique because it has been reported to be associated with better functional outcome and improved cost-effectiveness [23] in the early series, as compared to craniotomy or other MIN techniques. Per our clinical experience, MIN is considered appropriate for deep-seated ICH (i.e., >1 cm from the cortical surface), thalamic ICH, putaminal ICH, cerebellar ICH, or ICH with intraventricular hemorrhage (IVH) and hydrocephalus. Figure S1 shows a case with massive ICH for which the accompanying IVH was treated with minimally invasive endoscope-assisted ICH evacuation. For superficial or lobar ICH, craniotomy usually suffices and is straightforward. The selection of surgery was determined solely by the treating surgeon according to the clinical judgment.

All surgeries were performed within 8 h after the diagnosis of coagulopathy-related ICH to exclude the time to treat factor [18]. In our experience, where more than 84% of the cases were done within 4 h after onset, craniectomy was not sufficient to achieve decompression and ICP control [21].

### 2.3. MIN Technique

For most putaminal ICHs, we used the frontal burr hole approach to remove the clot [23,24]. In patients with thalamic ICH and IVH, the goal was to relieve the hydrocephalus and elevated ICP while removing the IVH and ICH as much as possible without causing further damage to the brain parenchyma. We therefore used the ipsilateral Kocher’s point as our entry point. Since the lateral ventricle was entered during surgery, an EVD was inserted through the operative tract. Bilateral approach with bilateral EVD placement might be considered in extensive IVH cases. Alternatively, using a more lateral trajectory, a septostomy might be done just like in the ventriculoscopic procedure to remove contralateral IVH [25].

Under general anesthesia, a linear skin incision (3–4 cm in length) was made depending on the chosen trajectory. A 1.5 cm burr hole was created with the dura opened. A 10 mm corticotomy was then done to insert the transparent plastic sheath (10 mm in outer diameter; length 5, 7, 9, or 12 cm depending on the depth of the hematoma) with the stylet. Real-time ultrasound guidance (Aloka UST-5268P-5 neurosurgery burr hole probe, 3.0–7.5 MHz, phased-array sector probe; Aloka, Tokyo, Japan) or navigation system might be helpful before the puncture step. After removing the stylet, the 4 mm 0° endoscope with irrigation system (18 cm in length; Karl Storz, Tuttlingen, Germany) was inserted into the sheath to provide visualization during ICH removal. We used the balanced suction irrigation technique to achieve maximal clot evacuation and inspect bleeders [26]. Local hemostatic agents were very useful in hemostasis during MIN for ICH [27]. If hemostatic agents did not stop the bleeding, the bleeder was identified using the balanced irrigation-suction technique, with constant irrigation and point suction. We usually used commercially available suction coagulation devices (11 Fr, 14 or 19 cm in length; Kirwan Surgical Products, Marshfield, MA, USA), which performed coagulation and suction simultaneously, to identify and cauterize the bleeder. After ICH removal and hemostasis, an ICP monitor was inserted as needed (according to the guideline, for pre-operative GCS ≤ 8 or herniation [28]) if the ventricle had not been inserted with an EVD.

### 2.4. Endpoints and Variables

Information on demographics, patient characteristics, medical history, coagulopathy etiology, medication use, preoperative ICH characteristics (i.e., initial Glasgow Coma Scale (GCS), admission ICH score, hematoma volume, presence of IVH), surgical records (i.e., surgical method, surgical duration, blood loss, hematoma clearance rate), and postoperative follow-up data (i.e., rebleeding, length of ICU stay, length of hospital stay, morbidity, GCS, Glasgow Outcome Scale Extended (GOSE), modified Rankin Scale (mRS)) was collected through the electronic medical record system. A higher GOSE score indicates a better functional outcome, while a higher mRS score implies a worse functional outcome. The volume of hematoma was calculated by the ABC/2 method (A: maximum length in axial cut of CT, B: width perpendicular to A on the same CT cut, and C: the number of slices multiplied by the slice thickness) [29].

The primary endpoints were 1-month rebleeding, morbidity, and mortality rates. The secondary endpoints were hematoma evacuation rate, surgical duration, blood loss, functional outcomes (i.e., GOSE and mRS), and length of ICU/hospital stay. Morbidity included wound dehiscence, surgical site infection, meningitis, ventriculitis, brain abscess, and sepsis. We collected postoperative GCS at 1 and 12 months and postoperative GOSE and mRS at 12 months.

### 2.5. Statistical Analysis

The data were first analyzed in a univariate fashion to compare the postoperative outcomes between craniotomy and MIN groups by the independent *t*-test or the Wilcoxon rank-sum test for continuous variables and the chi-square or Fisher’s exact test for categorical variables. Multivariate analysis was then performed to explore prognostic factors for postoperative outcomes. All statistical results were declared significant if *p* < 0.05. The analysis was performed using Statistical Analysis Software (SAS version 9.4, Cary, NC, USA).

## 3. Results

### 3.1. Patient Characteristics

**Figure 1:**
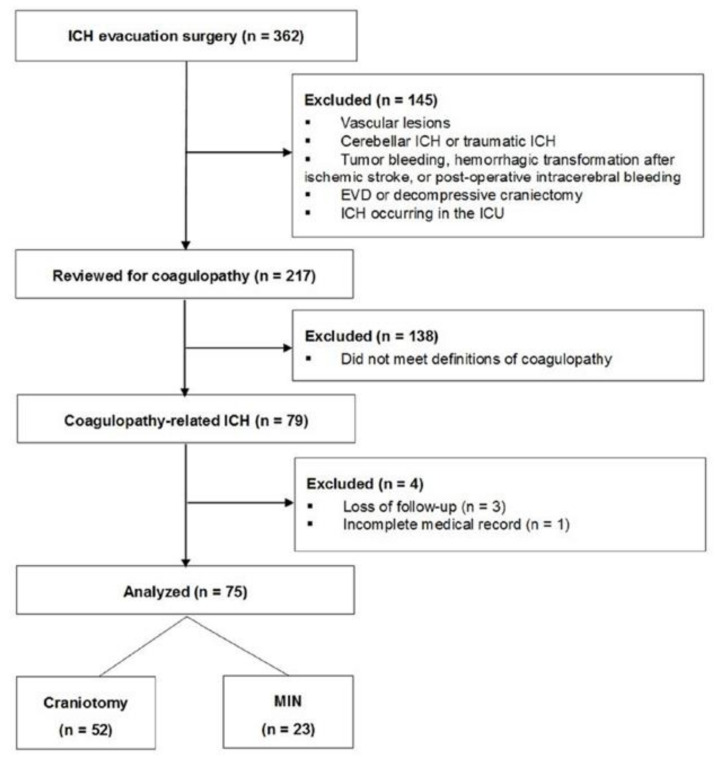
Patient disposition. Abbreviations: EVD, external ventricular drainage; ICH, intracerebral hemorrhage; ICU, intensive care unit; MIN, minimally invasive neurosurgery.

A total of 362 patients underwent surgery for ICH evacuation at NTUH between 2013 and 2018; 145 of these patients were disqualified due to exclusion criteria. Among the remaining 217 patients, we identified 79 eligible patients who fulfilled the definition of coagulopathy for this study. Four patients were excluded because of loss of long-term follow-up (*n* = 3) and incomplete medical records (*n* = 1), leaving 75 patients who were included in the analysis (Figure 1).

Among enrolled patients, 40 had deep ICH composed of 35 (87.5%) putaminal ICH and 5 (12.5%) thalamic ICH; the other 35 had lobar ICH, including 12 (34.3%) frontal lobar ICH, 5 (14.3%) temporal lobar ICH, 6 (17.1%) occipital lobar ICH, and 12 (34.3%) parietal lobar ICH.

Of 75 patients, 52 (33 had lobar ICH and 19 had deep ICH) underwent craniotomy and 23 (2 had lobar ICH and 21 had deep ICH) underwent MIN. There were no statistically significant between-group differences in demographics; clinical characteristics regarding coagulopathy such as the use of antithrombotic agents, thrombocytopenia, prolonged prothrombin time, and hemodialysis; or characteristics regarding index ICH such as admission GCS score, admission ICH score, hematoma volume, and the presence of IVH (Table 1). The mean ages in the craniotomy and MIN groups were 58.8 ± 17.61 and 60.5 ± 15.97 years, respectively. In the craniotomy group, the mean preoperative GCS score was 9.4 ± 4.06 and the mean preoperative ICH volume was 62.5 ± 27.76 mL; corresponding values in the MIN group were 9.4 ± 3.34 and 60.7 ± 24.47 mL, respectively. All 52 patients in the craniotomy group and 22 patients (95.7%) in the MIN group were administered antiplatelet agents or anticoagulants before the surgery. The antiplatelet agents included irreversible cyclooxygenase inhibitors (e.g., aspirin), adenosine diphosphate (ADP) receptor inhibitors (e.g., clopidogrel, ticlopidine), phosphodiesterase inhibitors (e.g., cilostazol), and adenosine reuptake inhibitors (e.g., dipyridamole); anticoagulants included VKAs and NOACs (e.g., dabigatran, rivaroxaban, apixaban).

### 3.2. Perioperative Records

Table 2 shows perioperative and postoperative outcomes for the MIN and craniotomy groups. The surgical time in the MIN group (mean, 121.8 ± 43.92 min) was significantly shorter than that in the craniotomy group (mean, 166.4 ± 63.82 min) (*p* = 0.003). The amount of surgical blood loss in the MIN group (mean, 69.6 ± 53.81 mL) was also significantly smaller than the amount in the craniotomy group (mean, 284.6 ± 368.56 mL) (*p* < 0.0001). There were no between-group differences in the length of ICU or hospital stays or hematoma clearance rates (all *p* > 0.05).

### 3.3. Postoperative Outcomes

The 1-month morbidity rate in the MIN group (8.7%) was significantly lower than that in the craniotomy group (30.8%) (*p* = 0.044). One-month mortality and rebleeding rates were not significantly different between the two groups, but there were more patients who expired or experienced rebleeding within 1 month in the craniotomy group than the MIN group (craniotomy vs. MIN: 19.2% vs. 8.7% for mortality, 23.1% vs. 4.4% for rebleeding).

As for functional outcomes, the 1-year GOSE score in the MIN group (4.0 ± 1.55) was significantly higher than that in the craniotomy group (3.1 ± 1.59) (*p* = 0.027). There were no significant between-group differences in postoperative GCS or mRS, but the mRS score in the MIN group (mean, 3.5 ± 1.34) was lower than that in the craniotomy group (mean, 4.1 ± 1.36), with borderline significance (*p* = 0.057).

Based on the multivariate linear regression analysis of 1-year functional outcomes (Table 3), MIN was independently associated with the improvement of GOSE (estimate = 0.99650, *p* = 0.0148) and mRS scores (estimate: −0.72849, *p* = 0.0427). The preoperative GCS score was found to be an independent predictor of good functional outcomes (1-year GOSE: estimate = 0.15563, *p* = 0.0451), while anticoagulant therapy was an independent predictor of poor functional outcome (1-year GOSE: estimate = −1.18483, *p* = 0.0043; 1-year mRS: estimate = 1.01080, *p* = 0.0059).

The multivariate logistic regression analysis of 1-month clinical events (mortality, rebleeding, and mortality) failed to demonstrate a significant effect for MIN compared with craniotomy (Table 4). Hematoma volume was the only independent predictor of postoperative death, rebleeding, and morbidity (Table 4).

### 3.4. Subgroup Analysis of Anticoagulant and Antiplatelet Agent Cohorts

Table 5 shows analyses for patients receiving anticoagulants and antiplatelet agents separately. Surgical blood loss in the MIN group was significantly less than that in the craniotomy group for both anticoagulant and antiplatelet agent cohorts. For the anticoagulant cohort, the 1-month morbidity rate in the MIN group (0.0%) was significantly lower than that in the craniotomy group (48.0%) (*p* = 0.029). There were no other significant differences in event rates between the craniotomy and MIN groups in both anticoagulant and antiplatelet agent cohorts; however, more patients in the craniotomy group died, or experienced rebleeding or morbidity within 1 month, compared with the MIN group.

In multivariable linear regression analysis, functional outcomes were better with basal ganglia ICH than with lobar ICH for the anticoagulant cohort, while ICH volume was negatively associated with functional outcomes for the antiplatelet agent cohort (Table 6 and Table 7). For both antithrombotic cohorts, multivariable regression analysis failed to reveal significant differences in outcomes according to type of surgery or antithrombotic agents (i.e., VKA or NOAC, clopidogrel, aspirin, or dual antiplatelet).

## 4. Discussion

The present study found that, in patients with coagulopathy-related ICH, MIN is safe and effective compared with craniotomy regarding postoperative outcomes. With its benefits of direct vision via image navigation and reduced surgical blood loss, our results revealed that MIN resulted in lower rates of death, rebleeding, and morbidity, as well as significant improvements in long-term functional outcomes, in comparison with craniotomy in this high-risk cohort.

Recent publications have described promising results with MIN, with decreased mortality and improved functional outcomes compared with traditional craniotomy [4,19,20,30,31,32]. Two meta-analyses have demonstrated that in selected ICH patients, MIN may be more beneficial than conventional medical treatment or craniotomy [18,33]. A previous study has also reported that endoscopic-assisted MIN was associated with better functional outcomes [23]. Since 2008, our hospital has amassed a dedicated ICH neurosurgical team. To date, we have performed MIN in more than 400 ICH patients and have significantly decreased mortality and improved functional outcomes in these patients [21,23,27]. The MIN approach generally evacuates ICH via stereotactic or endoscopic aspiration, with or without thrombolytic usage [28]. We have generally used the endoscopic-assisted method because it is faster for decompression and hemostasis can be achieved under direct visualization (as compared to other drainage procedures such as the MISTIE approach).

The pursuit of MIN in coagulopathy-related ICH patients is reasonable, since MIN avoids the inherent collateral damage due to brain retraction during traditional craniotomy, especially in deep-seated lesions, such as a putaminal or thalamic hemorrhage [34,35]. This is particularly important in coagulopathy-related ICH patients, as their vulnerable brain tissue is more prone to intraoperative bleeding or postoperative rebleeding. Such benefits were reflected in our study, which showed a lower rebleeding rate for MIN compared with craniotomy and an association of MIN with significant improvements in long-term functional outcomes. Our study also identified other benefits of MIN, including less blood loss, a shorter operative time, and faster recovery with shorter ICU and hospital stay, compared with craniotomy.

Mortality with anticoagulant-related ICH has been reported to be as high as 28–33% [14,15,16,17,36]. In the present study, mortality with anticoagulant-related ICH appeared to be lower in the MIN group than in the craniotomy group (14% vs. 36%). In the anticoagulant cohort, patients with deep-seated ICH were more likely to have favorable functional outcomes than those with lobar ICH, whereas the type of anticoagulant (NOAC or VKA) did not affect postoperative outcomes. In line with our results, three recent large-scale multicenter observational studies have suggested that baseline ICH volume, hematoma expansion, mortality, and functional outcomes were similar between NOAC- and VKA-related ICH [15,16,37]. Similarly, a cross-sectional survey involving 2245 Japanese patients has revealed no difference in surgical outcomes between VKA- and NOAC-related ICH groups [38]. For the antiplatelet agent cohort, our data suggest that a greater ICH volume may increase the likelihood of poor functional outcomes, yet the postoperative outcomes did not differ between the use of clopidogrel or aspirin. Considering that coagulopathy-related ICH patients may benefit more from MIN than craniotomy in functional outcomes, MIN may be more beneficial for certain coagulopathy patients at higher risk of functional disability, such as anticoagulant-treated patients, patients with poor GCS scores, and antiplatelet-treated patients with ICH expansion.

Besides the additional benefits of MIN in regard to long-term functional outcomes, our study sheds light on minimally invasive surgery for coagulopathy-related ICH by demonstrating that with meticulous perioperative medical management and well-selected surgical options, ICH can be evacuated safely and effectively from patients with coagulopathy. Several other studies have also revealed the benefits of a rapid reversal strategy as the perioperative management for coagulopathy-related ICH patients undergoing surgery to prevent hemorrhage expansion, limit tissue damage, and facilitate neurosurgical intervention [13,39,40].

Anticoagulant use and low initial GCS scores were found to be independent predictors of poor functional outcomes, whereas hemodialysis, antiplatelet treatment, thrombocytopenia, and the presence of IVH were not correlated with unfavorable functional outcomes or higher risks of postoperative death, rebleeding, and morbidity. Despite the MIN group having more patients with IVH and ICH scores of 4 or above than the craniotomy group, the surgical outcome was not compromised by these factors. Notably, several studies have reported that IVH can be evacuated more easily by MIN than by traditional craniotomy [8,41,42,43], which may explain why the presence of IVH was not a poor prognostic factor in our study. Moreover, all patients in our study were given prompt reversal of coagulopathy with 24 units of platelet transfusion during the perioperative period to correct the bleeding tendency. We assume that the reversal agents could correct these risk factors if they are used correctly.

This study is limited by its retrospective design, small sample size, and lack of generalizability, as data were collected from a single center. The retrospective design has the potential for informational bias, such as missing data and selection bias based on the surgeon’s clinical judgment. Indeed, the surgeon’s experience and preference as to the surgical plan were determinant factors for postoperative outcomes. In our study, all patients were operated on by the dedicated ICH neurosurgical team, which may have reduced biases from the surgeon’s practice. Despite these limitations, this is the first study as far as we know to investigate surgical outcomes of coagulopathy-related ICH and to determine the optimal surgical option for these patients by comparing MIN and craniotomy. It should be noted that postoperative outcomes following ICH evacuation vary widely across countries. ICH-related mortality is lower in Asia than in other countries [44]. Evidence also exists as to heterogeneity in efficacy and safety of NOACs and VKAs between Asian and non-Asian populations [45,46,47]. A multinational, randomized, controlled study that compares MIN and craniotomy for coagulopathy-related ICH is warranted.

## 5. Conclusions

Minimally invasive neurosurgery is safe and effective for coagulopathy-related ICH. It is associated with more favorable surgical and functional outcomes compared with traditional craniotomy. Further prospective, large-scale studies should be initiated to validate this promising result.

## Figures and Tables

**Table 1:** Baseline sociodemographic and clinical characteristics.

Variables	Craniotomy (*n* = 52)	MIN (*n* = 23)	*p*-Value
Age (year)	58.8 ± 17.61	60.5 ± 15.97	0.675
Male, *n* (%)	29 (55.8)	15 (65.2)	0.612
Anticoagulant, *n* (%)	25 (48.1)	7 (30.4)	0.207
Antiplatelet, *n* (%)	27 (51.9)	14 (60.9)	0.616
Thrombocytopenia, *n* (%)	9 (17.3)	3 (13.0)	0.745
Prolonged PT, *n* (%)	21 (40.4)	4 (17.4)	0.065
Hemodialysis, *n* (%)	3 (5.8)	3 (13.0)	0.363
GCS	9.4 ± 4.06	9.4 ± 3.34	0.895
IVH, *n* (%)	20 (38.5)	14 (60.9)	0.084
Hematoma volume (mL)	62.5 ± 27.76	60.7 ± 24.47	0.954
ICH score, *n* (%)			
1	9 (17.3)	4 (17.4)	0.428
2	21 (40.4)	6 (26.1)	
3	17 (32.7)	8 (34.8)	
4	4 (7.7)	5 (21.7)	
5	1 (1.9)	0 (0.0)	

Abbreviations: PT, prothrombin time; GCS, Glasgow Coma Scale; IVH, intraventricular hemorrhage; MIN, minimally invasive neurosurgery.

**Table 2:** Surgical and postoperative outcomes.

Outcome	Craniotomy (*n* = 52)	MIN (*n* = 23)	*p*-Value
Surgical duration (min)	166.4 ± 63.82	121.8 ± 43.92	0.003 *
Surgical blood loss (mL)	284.6 ± 368.56	69.6 ± 53.81	<0.0001 *
ICU stay (days)	14.8 ± 9.9	10.7 ± 5.71	0.162
Hospital stay (days)	39.6 ± 31.79	40.0 ± 12.11	0.642
Hematoma clearance rate (%)	77.1 ± 21.37	80.9 ± 16.02	0.913
Rebleeding, *n* (%)	12 (23.1)	1 (4.4)	0.054
Morbidity, *n* (%)	16 (30.8)	2 (8.7)	0.044 *
Mortality, *n* (%)	10 (19.2)	2 (8.7)	0.323
GCS on day 30	10.9 ± 4.17	11.3 ± 3.74	0.770
GCS at 1 year	10.6 ± 4.59	11.7 ± 3.87	0.438
GOSE at 1 year	3.1 ± 1.59	4.0 ± 1.55	0.027 *
mRS at 1 year	4.1 ± 1.36	3.5 ± 1.34	0.057

Abbreviations: ICU, intensive care unit; MIN, minimally invasive neurosurgery; GCS, Glasgow Coma Scale; GOSE, Glasgow Outcome Scale Extended; mRS, modified Rankin Scale. * Statistical significance.

**Table 3:** Multivariable linear regression analysis of functional outcomes at 1 year after surgery.

Variables	Estimate	SE	*t*	*p*-Value
Outcome: GOSE at 1 year				
Age	−0.00465	0.00997	−0.47	0.6423
Male	0.13788	0.33720	0.41	0.6841
Initial GCS	0.15563	0.07600	2.05	0.0451 *
Hematoma volume	−0.01230	0.00637	−1.93	0.0582
Anticoagulant	−1.18483	0.39865	−2.97	0.0043 *
Antiplatelet	−0.31453	0.38149	−0.82	0.4130
Hemodialysis	−0.65397	0.62699	−1.04	0.3013
Prolonged PT	0.86207	0.37226	2.32	0.0241 *
Thrombocytopenia	−0.16281	0.49824	−0.33	0.7450
IVH	−0.23827	0.56119	−0.42	0.6727
Lobar vs. deep ICH	−0.09549	0.39400	−0.24	0.8094
MIN vs. craniotomy	0.99650	0.39658	2.51	0.0148 *
Outcome: mRS at 1 year				
Age	0.00004444	0.00883	0.01	0.9960
Male	−0.28749	0.29886	−0.96	0.3401
Initial GCS	−0.12947	0.06736	−1.92	0.0595
Hematoma volume	0.00953	0.00564	1.69	0.0966
Anticoagulant	1.01080	0.35332	2.86	0.0059 *
Antiplatelet	0.30615	0.33812	0.91	0.3690
Hemodialysis	0.27753	0.55570	0.50	0.6194
Prolonged PT	−0.67103	0.32994	−2.03	0.0466 *
Thrombocytopenia	0.02441	0.44159	0.06	0.9561
IVH	0.27999	0.49739	0.56	0.5757
Lobar vs. deep ICH	0.09108	0.34920	0.26	0.7951
MIN vs. craniotomy	−0.72849	0.35149	−2.07	0.0427 *

Abbreviations: PT, prothrombin time; IVH, intraventricular hemorrhage; MIN, minimally invasive neurosurgery; GCS, Glasgow Coma Scale; GOSE, Glasgow Outcome Scale Extended; mRS, modified Rankin Scale; SE, standard error; ICH, intracerebral hemorrhage. * Statistical significance.

**Table 4:** Multivariable logistic regression analysis of clinical events at 1 month after surgery.

Variables	OR	SE	95% CI	*p*-Value
Outcome: 1-month mortality				
Age	0.950	0.0386	(0.881, 1.025)	0.1835
Male	0.157	1.5195	(0.008, 3.091)	0.2235
Initial GCS	0.793	0.2640	(0.472, 1.33)	0.3785
Hematoma volume	1.047	0.0231	(1, 1.095)	0.0487 *
Anticoagulant	37.845	1.7163	(1.309, >999.999)	0.0343 *
Antiplatelet	4.419	1.1068	(0.505, 38.675)	0.1794
Hemodialysis	29.367	2.3717	(0.281, >999.999)	0.1541
Prolonged PT	1.864	1.1342	(0.202, 17.214)	0.5830
Thrombocytopenia	0.464	1.5065	(0.024, 8.88)	0.6098
IVH	2.014	1.9021	(0.048, 83.769)	0.7129
Lobar vs. deep ICH	39.402	1.8910	(0.968, >999.999)	0.0520
MIN vs. craniotomy	3.322	1.9207	(0.077, 143.286)	0.5320
Outcome: 1-month rebleeding				
Age	1.011	0.0244	(0.964, 1.06)	0.6550
Male	0.492	0.8942	(0.085, 2.839)	0.4277
Initial GCS	1.046	0.1622	(0.761, 1.438)	0.7795
Hematoma volume	1.041	0.0156	(1.009, 1.073)	0.0106 *
Anticoagulants	1.759	0.9942	(0.251, 12.346)	0.5700
Antiplatelet	1.183	0.9136	(0.197, 7.089)	0.8541
Hemodialysis	<0.001	209.4	(<0.001, >999.999)	0.9601
Prolonged PT	0.535	0.8874	(0.094, 3.047)	0.4812
Thrombocytopenia	0.654	1.3535	(0.046, 9.29)	0.7541
IVH	0.818	1.2767	(0.067, 9.988)	0.8750
Lobar vs. deep ICH	1.330	0.9220	(0.218, 8.106)	0.7568
MIN vs. craniotomy	0.094	1.3541	(0.007, 1.337)	0.0809
Outcome: 1-month morbidity				
Age	0.978	0.0197	(0.941, 1.017)	0.2622
Male	0.893	0.7325	(0.212, 3.752)	0.8770
Initial GCS	1.108	0.1576	(0.814, 1.509)	0.5148
Hematoma volume	1.031	0.0143	(1.003, 1.061)	0.0307 *
Anticoagulants	5.803	0.9400	(0.92, 36.625)	0.0614
Antiplatelet	1.849	0.8598	(0.343, 9.975)	0.4745
Hemodialysis	0.764	1.4514	(0.044, 13.139)	0.8529
Prolonged PT	0.319	0.8072	(0.066, 1.554)	0.1575
Thrombocytopenia	0.886	1.1963	(0.085, 9.246)	0.9197
IVH	0.928	1.0791	(0.112, 7.694)	0.9449
Lobar vs. deep ICH	0.822	0.7969	(0.172, 3.919)	0.8056
MIN vs. craniotomy	0.199	0.9736	(0.03, 1.344)	0.0977

Abbreviations: PT, prothrombin time; IVH, intraventricular hemorrhage; MIN, minimally invasive neurosurgery; GCS, Glasgow Coma Scale; GOSE, Glasgow Outcome Scale Extended; mRS, modified Rankin Scale; OR, odds ratio; SE, standard error; CI, confidence interval. * Statistical significance.

**Table 5:** Surgical and postoperative outcomes of anticoagulant and antiplatelet agent cohorts.

Outcome	Anticoagulants			Antiplatelet Agent		
	Craniotomy (*n* = 25)	MIN (*n* = 7)	*p*-Value	Craniotomy (*n* = 27)	MIN (*n* = 14)	*p*-Value
Surgical duration (min)	167.7 ± 59.58	143.1 ± 42.55	0.362	166.3 ± 63.78	100.7 ± 32.44	<0.001 *
Surgical blood loss (mL)	338.0 ± 443.07	64.3 ± 24.40	0.006 *	255.6 ± 277.47	60.7 ± 21.29	<0.001 *
ICU stay (days)	16.3 ± 10.88	8.9 ± 2.97	0.156	16.2 ± 10.17	10.3 ± 6.28	0.093
Hospital stay (days)	34.9 ± 18.99	27.7 ± 11.04	0.351	45.1 ± 39.49	29.4 ± 12.81	0.386
Hematoma clearance rate (%)	64.4 ± 39.32	82.4 ± 11.21	0.494	73.9 ± 25.50	85.1 ± 7.30	0.475
Rebleeding, *n* (%)	8 (32.0)	0 (0.0)	0.150	7 (25.9%)	0 (0.0%)	0.075
Morbidity, *n* (%)	12 (48.0)	0 (0.0)	0.029 *	8 (29.6%)	1 (7.1%)	0.131
Mortality, *n* (%)	9 (36.0)	1 (14.3)	0.387	5 (18.5%)	2 (14.3%)	>0.999
GCS on day 30	9.7 ± 4.71	10.3 ± 4.46	0.782	10.6 ± 4.00	10.7 ± 4.10	0.801
GCS at 1 year	9.3 ± 5.30	10.3 ± 5.02	0.639	10.1 ± 4.53	11.0 ± 4.47	0.484
GOSE at 1 year	2.6 ± 1.55	3.6 ± 1.90	0.183	3.1 ± 1.67	3.7 ± 1.73	0.252
mRS at 1 year	4.5 ± 1.36	4.0 ± 1.53	0.412	4.1 ± 1.41	3.7 ± 1.49	0.354

Abbreviations: ICU, intensive care unit; MIN, minimally invasive neurosurgery; GCS, Glasgow Coma Scale; GOSE, Glasgow Outcome Scale Extended; mRS, modified Rankin Scale. * Statistical significance.

**Table 6:** Multivariable linear regression analysis of functional outcomes for anticoagulant and antiplatelet agent cohorts.

Variables	Anticoagulants				Antiplatelet Agent			
	Estimate	SE	*t*	*p*-Value	Estimate	SE	*t*	*p*-Value
Outcome: GOSE at 1 year								
Age	−0.02307	0.02023	−1.14	0.2719	−0.01130	0.01931	−0.59	0.5651
Male	0.22444	0.70776	0.32	0.7555	−0.31423	0.66402	−0.47	0.6414
Initial GCS	−0.01672	0.12771	−0.13	0.8976	0.10131	0.14283	0.71	0.4868
Hematoma volume	−0.01394	0.01157	−1.20	0.2470	-0.03190	0.01108	−2.88	0.0096 *
VKAs vs. NOACs	0.31520	0.82671	0.38	0.7084	--	--	--	--
Clopidogrel vs. aspirin	--	--	--	--	−1.64591	1.09649	−1.50	0.1498
Dual antiplatelet vs. aspirin	--	--	--	--	1.13290	0.92033	1.23	0.2334
Hemodialysis	−0.78686	1.72637	−0.46	0.6551	−1.53449	1.36579	−1.12	0.2752
Prolonged PT	0.66033	0.63047	1.05	0.3115	1.12662	0.74057	1.52	0.1447
Thrombocytopenia	1.11498	0.94553	1.18	0.2567	−2.48122	1.43867	−1.72	0.1008
IVH	0.72603	0.86924	0.84	0.4167	−0.54101	1.38124	−0.39	0.6997
Lobar vs. deep ICH	−1.67997	0.68970	−2.44	0.0278 *	0.62395	0.76093	0.82	0.4224
MIN vs. craniotomy	0.91136	0.88121	1.03	0.3174	1.23231	0.70757	1.74	0.0977
Outcome: mRS at 1 year								
Age	0.01383	0.01907	0.73	0.4796	0.00436	0.01695	0.26	0.7996
Male	−0.28969	0.66721	−0.43	0.6703	0.33443	0.58296	0.57	0.5729
Initial GCS	0.03788	0.12039	0.31	0.7573	−0.09280	0.12540	−0.74	0.4683
Hematoma volume	0.01357	0.01090	1.24	0.2325	0.02616	0.00973	2.69	0.0145 *
VKAs vs. NOACs	−0.33501	0.77934	−0.43	0.6734	--	--	--	--
Clopidogrel vs. aspirin	--	--	--	--	1.36025	0.96263	1.41	0.1738
Dual antiplatelet vs. aspirin	--	--	--	--	−0.93597	0.80798	−1.16	0.2611
Hemodialysis	0.91806	1.62745	0.56	0.5810	1.10433	1.19906	0.92	0.3686
Prolonged PT	−0.25648	0.59434	−0.43	0.6722	−1.15309	0.65016	−1.77	0.0922
Thrombocytopenia	−1.06294	0.89136	−1.19	0.2516	2.16372	1.26305	1.71	0.1030
IVH	−0.61882	0.81944	−0.76	0.4618	0.22619	1.21263	0.19	0.8540
Lobar vs. deep ICH	1.40155	0.65019	2.16	0.0478 *	−0.63281	0.66804	−0.95	0.3554
MIN vs. craniotomy	−0.08669	0.83072	−0.10	0.9183	−1.07239	0.62119	−1.73	0.1005

Abbreviations: PT, prothrombin time; IVH, intraventricular hemorrhage; MIN, minimally invasive neurosurgery; GCS, Glasgow Coma Scale; GOSE, Glasgow Outcome Scale Extended; mRS, modified Rankin Scale; SE, standard error; VKA, vitamin K antagonist; NOAC, novel oral anticoagulant. * Statistical significance.

**Table 7:** Multivariable logistic regression analysis of clinical events for anticoagulant and antiplatelet agent cohorts.

Variables	Anticoagulants				Antiplatelet Agent			
	OR	SE	95% CI	*p*-Value	OR	SE	95% CI	*p*-Value
Outcome: 1-month mortality								
Age	1.037	0.9644	(0.157, 6.868)	0.9696	0.711	3.3646	(0.001, 519.851)	0.9193
Male	<0.001	30.0929	(<0.001, >999.999)	0.2442	>999.999	116.7	(<0.001, >999.999)	0.8491
Initial GCS	0.853	7.1359	(<0.001, >999.999)	0.9822	415.377	13.4465	(<0.001, >999.999)	0.6539
Hematoma volume	1.877	0.5221	(0.674, 5.222)	0.2279	1.404	0.7660	(0.313, 6.303)	0.6574
VKAs vs. NOACs	69.362	69.7831	(<0.001, >999.999)	0.9516	--	--	--	--
Clopidogrel vs. aspirin	--	--	--	--	>999.999	180.1	(<0.001, >999.999)	0.9227
Dual antiplatelet vs. aspirin	--	--	--	--	>999.999	48.2435	(<0.001, >999.999)	0.4910
Hemodialysis	>999.999	140.9	(<0.001, >999.999)	0.5534	>999.999	81.6034	(<0.001, >999.999)	0.6108
Prolonged PT	5.917	13.5302	(<0.001, >999.999)	0.8955	>999.999	60.4025	(<0.001, >999.999)	0.8675
Thrombocytopenia	<0.001	55.0556	(<0.001, >999.999)	0.2236	>999.999	77.5521	(<0.001, >999.999)	0.6180
IVH	0.251	59.9477	(<0.001, >999.999)	0.9816	<0.001	105.2	(<0.001, >999.999)	0.7542
Lobar vs. deep ICH	>999.999	80.6885	(<0.001, >999.999)	0.2323	>999.999	87.4267	(<0.001, >999.999)	0.9140
MIN vs. craniotomy	>999.999	71.0683	(<0.001, >999.999)	0.8638	>999.999	97.5368	(<0.001, >999.999)	0.8786
Outcome: 1-month rebleeding								
Age	3.875	2.4295	(0.033, 453.208)	0.5772	1.018	1.4983	(0.054, 19.193)	0.9905
Male	<0.001	91.3900	(<0.001, >999.999)	0.3712	0.188	52.0048	(<0.001, >999.999)	0.9744
Initial GCS	>999.999	9.1731	(<0.001, >999.999)	0.3947	9.168	9.6752	(<0.001, >999.999)	0.8189
Hematoma volume	3.259	1.5559	(0.154, 68.78)	0.4477	1.287	0.9458	(0.202, 8.218)	0.7893
VKAs vs. NOACs	>999.999	178.1	(<0.001, >999.999)	0.6968	--	--	--	--
Clopidogrel vs. aspirin	--	--	--	--	0.141	91.4353	(<0.001, >999.999)	0.9829
Dual antiplatelet vs. aspirin	--	--	--	--	0.005	118.4	(<0.001, >999.999)	0.9637
Hemodialysis	>999.999	233.5	(<0.001, >999.999)	0.9122	817.945	123.9	(<0.001, >999.999)	0.9568
Prolonged PT	>999.999	19.2654	(<0.001, >999.999)	0.6306	0.860	123.2	(<0.001, >999.999)	0.9990
Thrombocytopenia	>999.999	127.1	(<0.001, >999.999)	0.8106	2.663	122.1	(<0.001, >999.999)	0.9936
IVH	<0.001	73.0805	(<0.001, >999.999)	0.4219	0.023	96.3720	(<0.001, >999.999)	0.9689
Lobar vs. deep ICH	>999.999	177.1	(<0.001, >999.999)	0.8967	0.646	60.9159	(<0.001, >999.999)	0.9943
MIN vs. craniotomy	>999.999	75.4084	(<0.001, >999.999)	0.9026	<0.001	62.3455	(<0.001, >999.999)	0.8159
Outcome: 1-month morbidity								
Age	0.293	1.3499	(0.021, 4.132)	0.3634	0.617	1.5948	(0.027, 14.058)	0.7622
Male	<0.001	61.0384	(<0.001, >999.999)	0.3363	>999.999	78.3938	(<0.001, >999.999)	0.8334
Initial GCS	126.463	7.0144	(<0.001, >999.999)	0.4902	320.953	12.7013	(<0.001, >999.999)	0.6496
Hematoma volume	3.332	1.2332	(0.297, 37.359)	0.3291	1.573	1.3047	(0.122, 20.288)	0.7285
VKAs vs. NOACs	>999.999	124.4	(<0.001, >999.999)	0.8893	--	--	--	--
Clopidogrel vs. aspirin	--	--	--	--	>999.999	110.1	(<0.001, >999.999)	0.8439
Dual antiplatelet vs. aspirin	--	--	--	--	>999.999	103.9	(<0.001, >999.999)	0.8643
Hemodialysis	>999.999	161.7	(<0.001, >999.999)	0.9072	>999.999	69.1641	(<0.001, >999.999)	0.5552
Prolonged PT	324.244	13.7150	(<0.001, >999.999)	0.6734	0.002	111.7	(<0.001, >999.999)	0.9553
Thrombocytopenia	<0.001	86.9442	(<0.001, >999.999)	0.7565	>999.999	96.8830	(<0.001, >999.999)	0.6754
IVH	<0.001	65.5522	(<0.001, >999.999)	0.5212	<0.001	300.3	(<0.001, >999.999)	0.9763
Lobar vs. deep ICH	240.791	92.8552	(<0.001, >999.999)	0.9529	<0.001	96.8247	(<0.001, >999.999)	0.6298
MIN vs. craniotomy	601.171	108.4	(<0.001, >999.999)	0.9529	<0.001	85.5689	(<0.001, >999.999)	0.6403

Abbreviations: PT, prothrombin time; IVH, intraventricular hemorrhage; MIN, minimally invasive neurosurgery; GCS, Glasgow Coma Scale; GOSE, Glasgow Outcome Scale Extended; mRS, modified Rankin Scale; OR, odds ratio; SE, standard error; CI, confidence interval; VKA, vitamin K antagonist; NOAC, novel oral anticoagulant.

## Data Availability

The data presented in this study are available on request from the corresponding author. The data are not publicly available due to ethical considerations.

## Supplementary Materials

The following are available online at https://www.mdpi.com/article/10.3390/life11060564/s1, Figure S1. A demonstration of a case with ICH accompanying IVH received minimally-invasive endoscope-assisted ICH evacuation.
