# Supplementary material for: Surgery for Coagulopathy-Related Intracerebral Hemorrhage: Craniotomy vs. Minimally Invasive Neurosurgery
Source: Life (Basel). 2021 Jun 15;11(6):564. doi: 10.3390/life11060564 (PMC8232628; doi:10.3390/life11060564)
Supplement: Supplementary file 1 [file life-11-00564-s001.zip › life-1236666-supplementary.pdf]

Article

Surgery for Coagulopathy-Related Intracerebral Hemorrhage: Craniotomy vs. Minimally Invasive Neurosurgery

Yen-Bo Liu [1,†], Lu-Ting Kuo [1,†], Chih-Hao Chen [2], Woon-Man Kung [3], Hsin-Hsi Tsai [2,4], Sheng-Chieh Chou [5], Shih-Hung Yang [1], Kuo-Chuan Wang [1], Dar-Ming Lai [1] and Abel Po-Hao Huang [1,*]

Supplementary materials

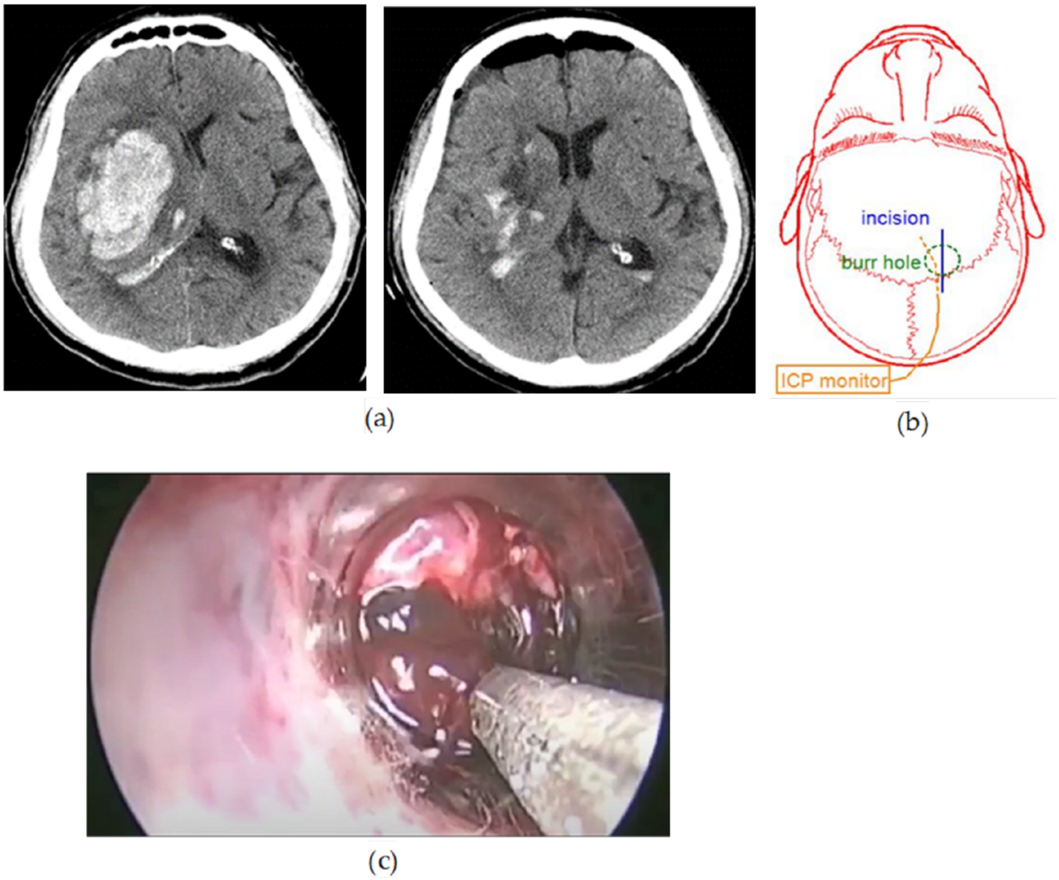

Figure S1. A case with ICH accompanying IVH received minimally-invasive endoscope-assisted ICH evacuation. **(a)** Pre- (left) and postoperative (right) head CT images; **(b)** a diagram showing a surgical position; **(c)** a screenshot during the evacuation.

A 68-year-old man who had coronary artery disease underwent off-pump coronary artery bypass (OPCAB) in 2012 and received aspirin in the meantime. He was unconscious on the street and was sent to our Emergency department right away. On arrival, his Glasgow Coma Scale (GCS) was E4M5V1. Neurological examination revealed left hemiplegia and preferential gaze to the right side. Emergent non-contrast head CT showed a massive ICH over right putamen with around 90 mL, causing a 12 mm midline shift, with the presence of intraventricular hemorrhage (IVH). The ICH score was 3 points, and surgical intervention was indicated. Therefore, we reversed the effect of aspirin by using 24 units of platelet transfusion. Then, we performed the surgery of minimally-invasive endoscope-assisted ICH evacuation with intracranial pressure (ICP) monitor insertion for this case. The surgery lasted for 113 minutes, and the

blood loss was 50 mL. After the surgery, the patient was admitted to the intensive care unit. Follow-up head CT image on postoperative day 1 showed a small amount of residual hematoma with an improvement of midline shift. His consciousness was improved gradually to E4M6V5, with left hemiparesis. We transferred him to an ordinary ward on postoperative day 5. After discharge, he kept rehabilitation and was followed up in the clinic regularly. At the 1-year postoperative follow-up, he could move his left limbs with antigravity.
